# Heat can erase epigenetic marks of vernalization in *Arabidopsis*

**DOI:** 10.4161/15592324.2014.990799

**Published:** 2015-02-03

**Authors:** Frédéric Bouché, Nathalie Detry, Claire Périlleux

**Affiliations:** University of Liège; Laboratory of Plant Physiology; PhytoSYSTEMS; Liège, Belgium

**Keywords:** *Arabidopsis*, devernalization, epigenetic modifications, *FLOWERING LOCUS C*, Flowering, H3K27me3, H3K4me3, vernalization

## Abstract

Vernalization establishes a memory of winter that must be maintained for weeks or months in order to promote flowering the following spring. The stability of the vernalized state varies among plant species and depends on the duration of cold exposure. In *Arabidopsis thaliana*, winter leads to epigenetic silencing of the floral repressor gene *FLOWERING LOCUS C* (*FLC*) and the duration of cold is measured through the dynamics of chromatin modifications during and after cold. The growing conditions encountered post-vernalization are thus critical for the maintenance of the vernalized state. We reported that high temperature leads to devernalization and, consistently, to *FLC* reactivation in *Arabidopsis* seedlings. Here we show that the repressive epigenetic mark H3K27me3 decreases at the *FLC* locus when vernalized seedlings are grown at 30°C, unless they were first exposed to a stabilizing period at 20°C. Ambient temperature thus controls the epigenetic memory of winter.

Vernalization-requiring plants are unable to flower or flower very late unless they are exposed to prolonged cold temperatures. The ecological significance of this trait is to block flowering prior to winter and permit the floral transition under the favorable conditions of following spring, when ambient temperature and daylength increase.^1^ In *Arabidopsis thaliana*, vernalization requirement is largely due to the high expression level of the *FLOWERING LOCUS C* (*FLC*) gene encoding a MADS-domain protein.^2,3^ FLC acts as a transcriptional inhibitor of the flowering integrator genes *FLOWERING LOCUS T* (*FT*) and *SUPPRESSOR OF OVEREXPRESSION OF CO 1* (*SOC1*).^4-7^ Vernalization leads to stable silencing of *FLC* and so relieves *FT* and *SOC1,* which can then be activated by a range of environmental or endogenous signals stimulating flowering (reviewed in refs. 8–12).

The initial level of *FLC* expression depends on the balance between activation and repression activities of *FRIGIDA* (*FRI*) and the autonomous flowering pathway, respectively, which regulate antisense *FLC* transcripts (called COOLAIR) and histone modifications (reviewed in ref. 13). Allelic variation at *FRI* and *FLC* loci largely explain the differences in vernalization requirement between winter and summer accessions of *Arabidopsis.**^14-18^* Prior to vernalization, histone modifications associated with actively transcribed genes, such as H3K4me3 and H3K36me2/me3, are enriched at *FLC* whereas repressive marks such as H3K27me3 are present but low abundant.^19-24^ During vernalization, gradual accumulation of the repressive mark H3K27me3 occurs at the nucleation region, just downstream of the transcription initiation site, and thereafter spreads over the whole region of *FLC* upon return to warm temperature.^19,25-27^

In *Arabidopsis* as well as some other species such as Henbane, vernalization is both quantitative – short cold spells have no effect on flowering – and stable, since cold exposure and the subsequent floral transition may be separated by several months.^8^ There are, however, species in which vernalization is not stable.^9,28^ In species with a stable vernalized state, the memory of cold is only maintained after long cold exposure, and this behavior reflects the quantitative nature of the response. At the molecular level, it has been shown that the accumulation of H3K27me3 during vernalization reflects the length of the cold exposure and cell-autonomously regulates the transition from an ‘active’ to a ‘silenced’ state of *FLC.**^25^* After longer cold treatment, an increasing number of cells are switched off and thus the overall *FLC* level is gradually turned down^29^ and reflects the average state of a cell population.^25^ Then, upon return to warm temperature, fast deposition of repressive histone marks across the whole gene allows and is required for maintenance of the silenced state,^30^ which is limited to mitotically active tissues.^31^

At the physiological level though, the stability of vernalization does not only depend on its duration, but also on the growing conditions prevailing post-vernalization.^32^ ‘Warm temperatures’ - as used in experimental work on *Arabidopsis* - are around 20°C and stabilize the vernalized state. By contrast, ‘hot temperatures’ can devernalize the plants, as reported in early work on *Arabidopsis.*^33,34^ We showed previously that one week at 30°C given directly after a saturating vernalization period completely suppressed the vernalized state and reactivated *FLC* in *Arabidopsis.*^28^ By contrast, the same treatment given after 2 ‘stabilizing’ weeks of 20°C had no effect. As a complementary experiment, we analyzed the effect of one week at 30°C on the chromatin mark profiles at the *FLC* locus.

ChIP analyses were performed on high-expressing *FLC* seedlings (Col-FRI)^35^ harvested 3 weeks after the end of a 6-week vernalization period or a 3-day stratification as a control (**Fig. 1**). Protocols for chromatin extraction and use of anti-H3K27me3 and -H3K4me3 antibodies followed manufacturer's instructions (www.diagenode.com).

**Figure 1.**
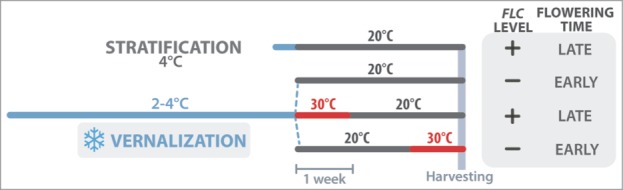
Experimental set-up. Col-FRI seeds were either stratified for 3 days at 4°C or vernalized for 6 weeks at 2–4°C. After the cold period, seedlings were transferred to 20°C (10:14 h light:dark cycles, 100 μE.m^−2^.s^−1^) or were first exposed to a devernalization treatment of 1 week at 30°C before transfer to 20°C conditions. The effects of these different post-vernalization conditions on *FLC* transcript level and flowering are summarized on the right according to Périlleux et al.^28^

We observed that the repressive H3K27me3 marks were much more abundant in vernalized than in non-vernalized seedlings (**Fig. 2**), and distributed across the *FLC* gene as previously reported.^25,26^ By contrast, vernalization had a very weak effect on the abundance of the H3K4me3 activation marks (**Fig. 3**). Most interestingly, the 30°C treatment given straight after vernalization completely prevented the accumulation of H3K27me3 marks at all the tested regions of the *FLC* gene, including the nucleation site (regions I to III in **Fig. 2**). A slight increase in H3K4me3 activation marks was also detected (**Fig. 3**). These results are fully consistent with the fact that this 30°C treatment reactivates *FLC* and inhibits flowering^28^ (**Fig. 1**). We can thus conclude that heat removes the epigenetic marks of vernalization when it occurs straight after cold. By contrast, when the heat treatment was given after 2 weeks at 20°C, the repressive H3K27me3 marks of vernalization remained on the chromatin and the vernalized state was maintained (**Fig. 2**). Angel et al.^25^ evaluated to 3 days the time taken after cold to achieve maximum H3K27me3 deposition at *FLC* locus, and hence the growing conditions occurring then are critically important for maintenance of the vernalized state. The erasing effect of heat (**Fig. 4**) highlights a risk for winter plants if we think of the extreme and erratic weather events that are becoming more common as the climate changes.

**Figure 2.**
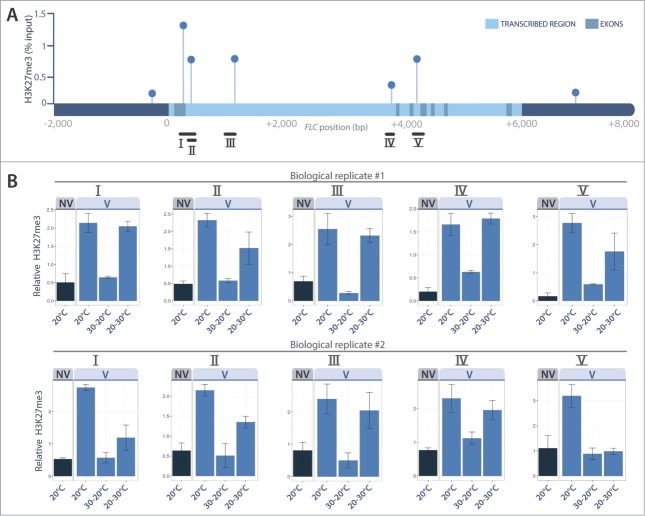
H3K27me3 ChIP experiments. H3K27me3 profile across *FLC*, for non vernalized seedlings (NV) and after 6 weeks of vernalization (V) followed by 3 weeks of warm (20°C), or 1 week of heat (30°C) given before (30–20°C) or after (20–30°C) 2 weeks of warm. (**A**) H3K27me3 profile expressed as % of chromatin input retrieved for the V/20°C sample. (**B**) H3K27me3 quantifications (**±** SE of 3 technical replicates) relative to constitutive marks at *AGAMOUS* and *SHOOT MERISTEMLESS* genes. Two independent biological replicates are shown; similar results were obtained in a third experiment (Supplemental **Fig. S1**). Primers used are listed in **Table S1**.

**Figure 3.**
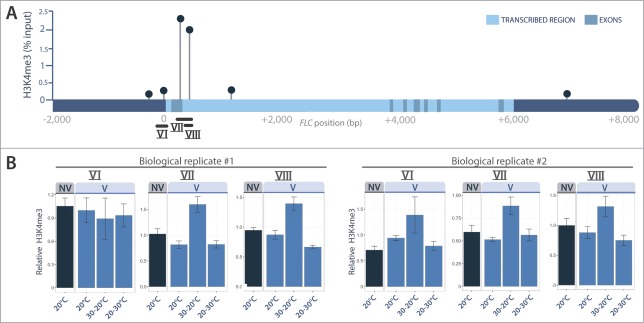
H3K4me3 ChIP experiments. H3K4me3 profile across *FLC*, for non vernalized seedlings (NV) and after 6 weeks of vernalization (V) followed by 3 weeks of warm (20°C), or 1 week of heat (30°C) given before (30–20°C) or after (20–30°C) 2 weeks of warm. (**A**) H3K4me3 profile expressed as % of chromatin input retrieved for the NV/20°C sample. (**B**) H3K4me3 quantifications (**±** SE of 3 technical replicates) relative to constitutive marks at *ACTIN7* and *UBIQUITIN10* genes. Two independent biological replicates are shown. Primers used are listed in **Table S2**.

**Figure 4.**
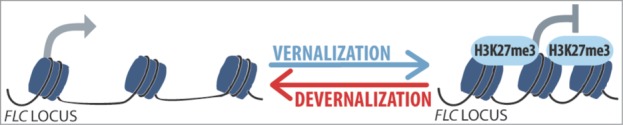
Model of the devernalization process. Vernalization represses the expression of *FLC* through chromatin modifications, mainly an increase in H3K27me3. Heat occurring just after vernalization restores *FLC* activity and this is at least partly due to the removal of H3K27me3 repressive marks.

## Supplemental Material

Primers are listed in **Tables S1** and **S2**. Supplemental **Fig. S1** shows a third biological replicate of H3K27me3 analyses.
