# Supplementary material for: Heat can erase epigenetic marks of vernalization in Arabidopsis
Source: Plant Signal Behav. 2015 Feb 3;10(3):e990799. doi: 10.4161/15592324.2014.990799 (PMC4622702; doi:10.4161/15592324.2014.990799)
Supplement: Figure_S1.docx [file kpsb-10-03-990799-s003.docx]

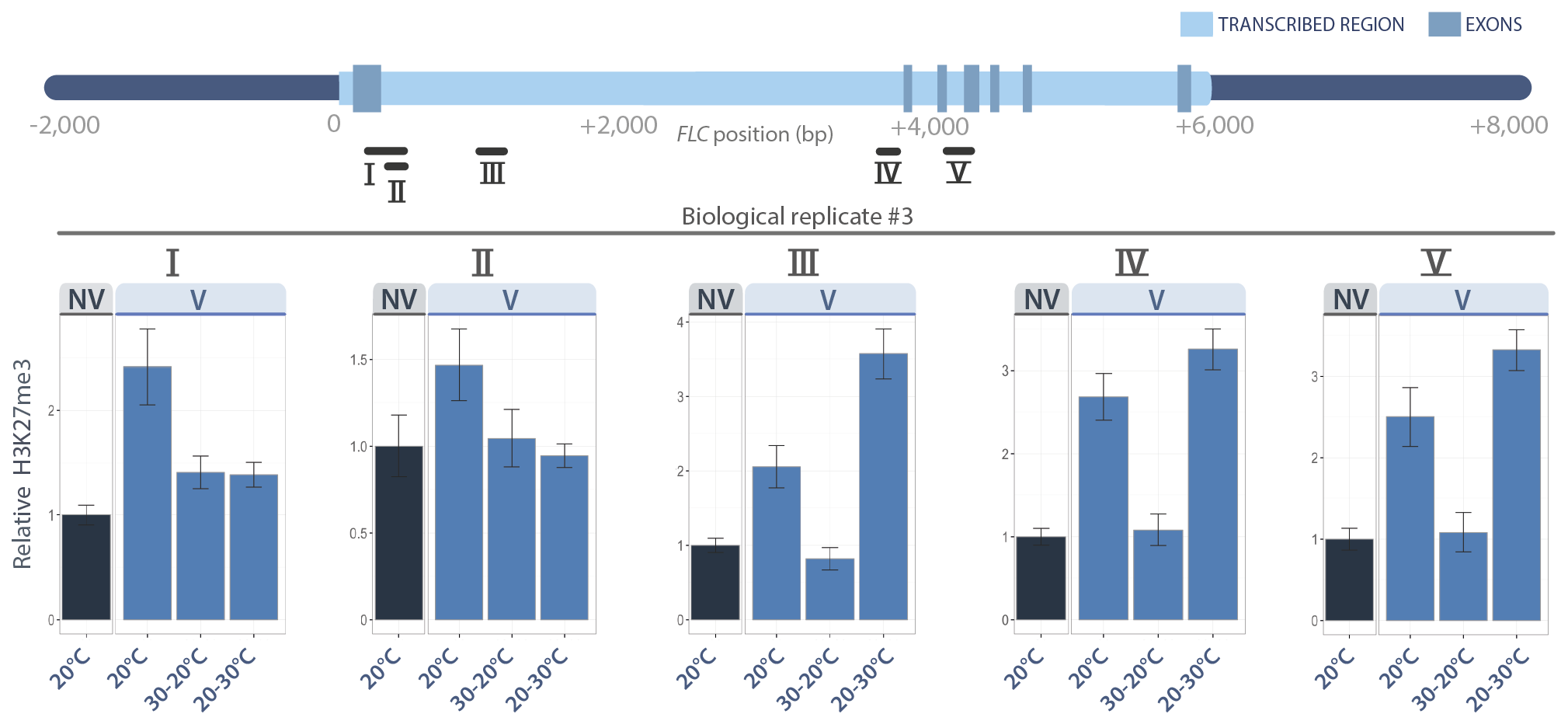


**Figure S1. Biological replicate of H3K27me3 experiment.** H3K27me3 relative abundance (**±** SE of 3 qPCR technical replicates) at different positions of the *FLC* locus for non vernalized seedlings (NV) and after 6 weeks of vernalization (V) followed by 3 weeks of warm (20°C), or 1 week of heat (30°C) given before (30-20°C) or after (20-30°C) 2 weeks of warm. H3K27me3 quantifications are relative to constitutive marks at *AGAMOUS* and *SHOOT MERISTEMLESS* genes. Primers used are listed in table S1.
